# Long-term clinical effects of epalrestat, an aldose reductase inhibitor, on progression of diabetic neuropathy and other microvascular complications: multivariate epidemiological analysis based on patient background factors and severity of diabetic neuropathy

**DOI:** 10.1111/j.1464-5491.2012.03684.x

**Published:** 2012-11-14

**Authors:** N Hotta, R Kawamori, M Fukuda, Y Shigeta

**Affiliations:** 1 Chubu Rosai HospitalNagoya; 2 Sportology Centre, Juntendo University Graduate School of MedicineTokyo; 3 NTT West Osaka HospitalOsaka; 4 Shiga University of Medical ScienceOtsu, Japan

## Abstract

**Aims:**

The goal of the study was to evaluate the efficacy of epalrestat, an aldose reductase inhibitor, on diabetic retinopathy and diabetic nephropathy, based on analysis of the results of the Aldose Reductase Inhibitor–Diabetes Complications Trial, a 3-year multicentre comparative clinical trial of conventional therapy (control group) and epalrestat therapy (epalrestat group) in Japanese patients with mild diabetic neuropathy.

**Methods:**

The subjects of the study were patients enrolled in the Aldose Reductase Inhibitor–Diabetes Complications Trial for whom data for major patient characteristics, severity of diabetic neuropathy at the end of the study and time-courses of diabetic retinopathy and diabetic nephropathy were available (57 and 52 patients from the control and epalrestat groups, respectively). Progression of diabetic retinopathy/nephropathy (a primary endpoint) in relation to major patient characteristics, severity of diabetic neuropathy at the end of the study (assessed from the mean of *z*-scores in four neurological function tests) and epalrestat treatment were analysed using univariate analysis and multiple logistic regression analysis.

**Results:**

Progression of diabetic retinopathy/nephropathy was significantly inhibited in the epalrestat group compared with the control group (odds ratio = 0.323, *P* = 0.014) and was dependent on the severity of diabetic neuropathy at the end of the study (odds ratio = 2.131, *P* = 0.025).

**Conclusions:**

Epalrestat prevented progression of diabetic neuropathy and retinopathy/nephropathy. The effect on diabetic retinopathy/nephropathy may have occurred indirectly because of the prevention of progression of diabetic neuropathy, in addition to the inhibitory action of epalrestat on aldose reductase.

## Introduction

Diabetic neuropathy has a high incidence and is associated with a risk of foot ulcer, amputation, gastroparesis, genitourinary tract disorder, cardiovascular disease and erectile dysfunction [1–3]. Moreover, diabetic neuropathy is strongly associated with diabetic retinopathy/nephropathy [1,3–5]. Previously, we conducted the Aldose Reductase Inhibitor-Diabetes Complications Trial, a 3-year multicentre comparative clinical trial of conventional therapy (control group) and epalrestat, an aldose reductase inhibitor, with conventional therapy (epalrestat group) in Japanese patients with mild diabetic neuropathy. Epalrestat was found to be effective for both diabetic neuropathy and for early retinopathy [6–8]. In the present study, the Aldose Reductase Inhibitor-–Diabetes Complications Trial results were re-analysed to examine the effect of epalrestat on diabetic retinopathy/nephropathy in more detail.

## Patients and methods

The Aldose Reductase Inhibitor-–Diabetes Complications Trial methodology has been described previously [6]. The protocol was approved by the Institutional Review Board of each medical facility and all patients gave informed consent.

The subjects in the current study (control group *n* = 57; epalrestat group *n* = 52) were selected from patients in the Aldose Reductase Inhibitor-–Diabetes Complications Trial for whom data for major patient characteristics, neurological function tests at the end of the study, retinal findings and an evaluation of nephropathy were available. Epalrestat (50 mg) was administered orally three times daily before each meal (150 mg/day). The primary endpoint was the presence of progression of diabetic retinopathy/nephropathy. The major patient characteristics were age (< 60 years, 60 to < 70 years, ≥ 70 years), duration of diabetes (< 10 years, ≥ 10 years), BMI (< 25 kg/m^2^, ≥ 25 kg/m^2^), baseline HbA_1c_ [< 57 mmol/mol (7.4%), ≥ 57 mmol/mol (7.4%)], HbA_1c_ over the 3-year period of the study [< 57 mmol/mol (7.4%), ≥ 57 mmol/mol (7.4%) to < 79 mmol/mol (9.4%), ≥ 79 mmol/mol (9.4%)], presence/absence of hypertension, and presence/absence of hyperlipidaemia. International Federation of Clinical Chemistry and Laboratory Medicine HbA_1c_ values (mmol/mol) were calculated from National Glycohaemoglobin Standardization Programme units (%) using the online HbA_1c_ converter (*Diabetic Medicine* author guidelines). National Glycohaemoglobin Standardization Programme units were calculated as Japan Diabetes Society units (%) + 0.4 (%) [9]. International Federation of Clinical Chemistry units are listed first, followed by National Glycohaemoglobin Standardization Programme units in parentheses.

Data were standardized for four neurological function test parameters (median motor nerve conduction velocity, minimum F-wave latency of the median motor nerve, vibration perception threshold and coefficient of variation of the R-R interval at rest (CV_R-R_)] at the end of the study and the *z*-scores for the data were calculated [10]. The mean of the *z*-scores for the four parameters, reflecting the severity of diabetic neuropathy in each patient, was divided into quartiles, and severity was classified into four stages (least, 27 patients; slight, 27 patients; moderate, 27 patients; and severe, 28 patients).

Diabetic retinopathy was evaluated by an ophthalmologist in a blinded manner based on retinal findings (fundus photographs) over the 3-year period of the study. Progression was defined as new development of retinopathy, such as punctate haemorrhages and hard exudates, progression of findings, such as soft exudates/neovascularization, or laser photocoagulation [11]. Diabetic nephropathy was classified into three stages based on diagnostic criteria for microalbuminuria of the American Diabetes Association: normal, microalbuminuria and clinical albuminuria [12]. Findings were compared between baseline and 3 years later to determine if progression had occurred.

### Statistical analysis

The presence of progression of diabetic retinopathy/nephropathy was analysed in relation to patient background factors, severity of diabetic neuropathy at the end of the study and epalrestat treatment using univariate analysis and multiple logistic regression analysis. In univariate analysis, a χ^2^ test was used to evaluate the significance of the effects of age, duration of diabetes, BMI, baseline HbA_1c_, HbA_1c_ over the 3 years of the study, hypertension, hyperlipidaemia, and epalrestat treatment; a Cochran–Armitage trend test was used for severity of diabetic neuropathy. In multiple logistic regression analysis, a Wald test was used to test the significance of the coefficient of each factor. A *P*-value < 0.05 was considered significant in all analyses.

## Results

Univariate analysis showed that no background factor was significantly related to the progression of diabetic retinopathy/nephropathy, but the proportion of patients with progression tended to increase as the severity of diabetic neuropathy increased (*P* = 0.066). Progression of diabetic retinopathy/nephropathy was significantly lower in the epalrestat group (20 patients, 38.5%) compared with the control group (33 patients, 57.9%) (*P* = 0.043) (Table 1).

**Table 1:** Effects of background factors and epalrestat on progression of diabetic retinopathy/nephropathy

Factors	Number of patients	Diabetic retinopathy/nephropathy		*P*
Progression, *n* (%)	Improvement/no change, *n* (%)
Age, years				
< 60	37	16 (43.2)	21 (56.8)	0.664*
≥ 60 to < 70	51	27 (52.9)	24 (47.1)
≥ 70	21	10 (47.6)	11 (52.4)
Duration of diabetes, years				
< 10	43	20 (46.5)	23 (53.5)	0.722*
≥ 10	66	33 (50.0)	33 (50.0)
BMI, kg/m^2^				
< 25	72	35 (48.6)	37 (51.4)	0.997*
≥ 25	37	18 (48.6)	19 (51.4)
Baseline HbA_1c_, mmol/mol (%)				
< 57 (7.4)	50	22 (44.0)	28 (56.0)	0.374*
≥ 57 (7.4)	59	31 (52.5)	28 (47.5)
HbA_1c_ over 3 years, mmol/mol (%)				
< 57 (7.4)	20	10 (50.0)	10 (50.0)	0.605*
≥ 57 (7.4) to < 79 (9.4)	74	34 (45.9)	40 (54.1)	
≥ 79 (9.4)	15	9 (60.0)	6 (40.0)
Hypertension				
No	59	25 (42.4)	34 (57.6)	0.156*
Yes	50	28 (56.0)	22 (44.0)
Hyperlipidaemia				
No	73	35 (47.9)	38 (52.1)	0.840*
Yes	36	18 (50.0)	18 (50.0)
Standardized severity of diabetic neuropathy				
Least	27	11 (40.7)	16 (59.3)	0.066†
Slight	27	11 (40.7)	16 (59.3)	
Moderate	27	13 (48.1)	14 (51.9)	
Severe	28	18 (64.3)	10 (35.7)
Epalrestat				
No	57	33 (57.9)	24 (42.1)	0.043*
Yes	52	20 (38.5)	32 (61.5)

The standardized severity of diabetic neuropathy obtained from four nerve function parameters [median motor nerve conduction velocity, minimum F-wave latency of the median motor nerve, vibration threshold, and coefficient of variation of the R-R interval at rest (CV_R-R_)] was classified into four stages from least to severe by quartiles. The details are described in the Patients and Methods section.

*χ^2^ test; †Cochrane–Armitage trend test.

**Figure 1:**
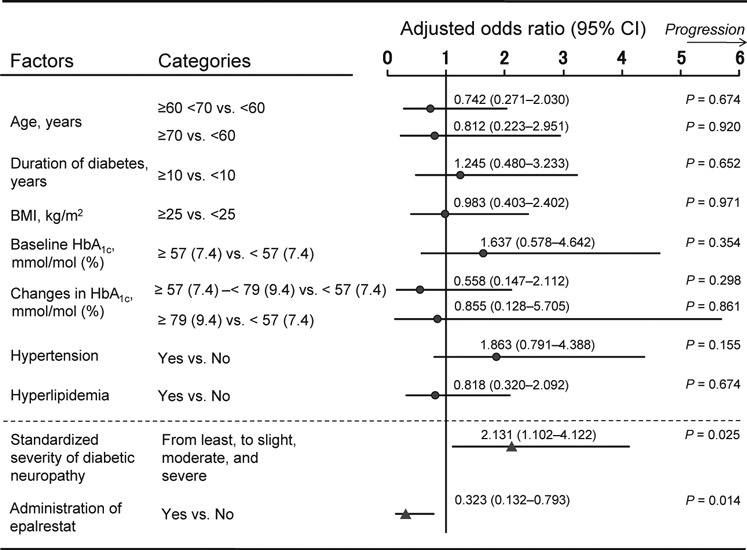
Multiple logistic regression analysis of the efficacy of epalrestat and the effect of patient background on progression of diabetic retinopathy/nephropathy. The standardized severity of diabetic neuropathy obtained from four nerve function parameters [median motor nerve conduction velocity, minimum F-wave latency of the median motor nerve, vibration perception threshold and coefficient of variation of the R-R interval at rest (CV_R-R_)] was classified into four stages from least to severe by quartiles. The details are described in the Patients and Methods section. A Wald test was performed to evaluate the significance of each factor.

In multiple logistic regression analysis of the progression of diabetic retinopathy/nephropathy, the odds ratio for the effect of severity of diabetic neuropathy was 2.131 (95% confidence interval 1.102–4.122; *P* = 0.025) and that for epalrestat treatment compared with the control group was 0.323 (95% confidence interval 0.132–0.793; *P*= 0.014). These results show that diabetic retinopathy/nephropathy progressed as the severity of diabetic neuropathy increased and that epalrestat suppressed this progression (Fig. 1).

## Discussion

Complications such as neuropathy, retinopathy, and nephropathy are problematic in patients with diabetes mellitus. Large-scale studies such as the Diabetes Control and Complications Trial and the United Kingdom Prospective Diabetes Study have shown that onset or progression of these complications can be suppressed to some degree if glycaemic control is maintained [13–16]. However, even with intensive glycaemic control, these complications cannot be completely prevented [13–16]. To prevent the onset/progression of these complications, treatment based on their pathogenesis may be useful. Increased activity of the polyol pathway is an important pathogenic factor in diabetic complications [3,8,17] and several inhibitors of aldose reductase, a key enzyme in this pathway, have been developed. However, epalrestat is the only aldose reductase inhibitor that is currently available for clinical use in Japan [18–21].

In the current study, the efficacy of epalrestat for diabetic retinopathy/nephropathy was examined by re-analysis of the results of the Aldose Reductase Inhibitor–Diabetes Complications Trial, with consideration of the influence of patient background factors and severity of diabetic neuropathy. The results confirmed that epalrestat significantly suppresses the progression of diabetic retinopathy/nephropathy. Moreover, the milder the severity of diabetic neuropathy at the end of the study, the greater the suppression of the progression of diabetic retinopathy/nephropathy, which was an interesting finding.

The Aldose Reductase Inhibitor–Diabetes Complications Trial was started in patients with mild diabetic neuropathy [6–8] and this condition remained mild in most patients over the 3-year period of the study. Among these patients, those who received epalrestat treatment also showed reduced development of diabetic retinopathy/nephropathy, which may have resulted from the suppressive effect of epalrestat on oxidative and inflammatory stress through inhibition of the polyol pathway [8]. However, we cannot exclude the possibility that maintenance of mild diabetic neuropathy may also have prevented progression of diabetic retinopathy/nephropathy. Consistent with this possibility, Barr *et al.* [22] reported that in a population-derived sample of individuals with impaired glucose tolerance or impaired fasting glucose, those with neuropathy were nearly four times more likely to have retinopathy and two times more likely to have albuminuria compared with those without neuropathy. Kärvestedt *et al.* [23] also found that the prevalence of peripheral sensory neuropathy increased with the severity of retinopathy. Thus, diabetic neuropathy, diabetic retinopathy and diabetic nephropathy, which are all microvascular complications, may be mutually and closely related, with diabetic neuropathy acting as a possible trigger for the onset or progression of the other complications. Indeed, Charles *et al.* [24] found evidence that low peripheral nerve conduction velocities and amplitudes are strongly related to diabetic microvascular complications in Type 1 diabetes.

Increased activity of protein kinase Cβ is thought to be of importance in the mechanism of diabetic retinopathy/nephropathy caused by hyperglycaemia [3,8,21,25]. Thus, hyperglycaemia promotes the activation of protein kinase Cβ, and diabetic retinopathy/nephropathy develops and progresses through activation of vascular endothelial growth factor and accumulation of extracellular matrix and activation of transforming growth factor-β [8,18,26,27]. We have previously shown that incubation of rat aortic smooth muscle cells in the presence of a high glucose concentration resulted in significant increases of protein kinase C activity and expression of the protein kinase C βII isoform, and that these increases were suppressed by epalrestat [28]. In a study of human coronary artery smooth muscle cells, Yasunari *et al.* [29] also found that epalrestat inhibited an increase in membrane-bound protein kinase C. These reports address macroangiopathies, but we have also found that an aldose reductase inhibitor reduced high glucose-induced apoptosis in cultured bovine retinal pericytes [30], and Gerhardinger *et al.* [31] showed that an aldose reductase inhibitor inhibited up regulation of genes in the transforming growth factor-β pathway and apoptosis in retinal vessels of diabetic rats. Therefore, increased activity of the polyol pathway may also be closely related to increased activity of protein kinase Cβ and transforming growth factor-β in microangiopathies.

In the first report of the Aldose Reductase Inhibitor–Diabetes Complications Trial the epalrestat and control groups had 289 and 305 subjects, respectively [6], but in this study these numbers were 52 and 57, respectively. Fewer subjects were included because assessment of diabetic retinopathy/nephropathy was not used as a primary endpoint. However, the distribution of background factors in the two groups in the current study (data not shown) was similar to that in the earlier report [6]. There are some limitations because of the open-label trial design but bias was minimized because the nerve function tests, electromyogram and retinopathy assessments were performed under blinded conditions [6,7].

In the trial [6–8], epalrestat was shown to suppress onset/progression of both diabetic neuropathy and diabetic retinopathy/nephropathy over a 3-year period. Moreover, reduced progression of diabetic retinopathy/nephropathy was found in patients with milder diabetic neuropathy. It is unclear if the prevention of progression of diabetic retinopathy/nephropathy is results from inhibition of aldose reductase by epalrestat or a secondary effect because of maintenance of milder diabetic neuropathy, or a combination of both. No other risk factors for onset/progression of diabetic retino-pathy/nephropathy were found in the current study, although a tendency for an association with hypertension (*P* = 0.155) was observed and might be clarified in a larger-scale study. It has been proposed that angiotensin-converting enzyme inhibitors and angiotensin receptor blockers can influence the progression of nephropathy/retinopathy. Angiotensin-converting enzyme inhibitors/angiotensin receptor blockers were given alone or in combination in the Aldose Reductase Inhibitor-–Diabetes Complications Trial (medication rate, 25–50%), but there was no significant difference in medication rate between the control and epalrestat groups.

In conclusion, treatment with epalrestat at an early stage is effective for delaying progression of diabetic neuropathy and can also prevent the onset/progression of diabetic retinopathy/nephropathy. Therefore, epalrestat is a clinically effective drug that may also be beneficial in reducing medical costs.
